# Cdc42 and RhoA reveal different spatio-temporal dynamics upon local stimulation with Semaphorin-3A

**DOI:** 10.3389/fncel.2015.00333

**Published:** 2015-08-26

**Authors:** Federico Iseppon, Luisa M. R. Napolitano, Vincent Torre, Dan Cojoc

**Affiliations:** ^1^Neurobiology Sector, International School for Advanced StudiesTrieste, Italy; ^2^Structural Biology Laboratory, Elettra-Sincrotrone Trieste S.C.p.A.Trieste, Italy; ^3^Institute of Materials – National Research CouncilTrieste, Italy

**Keywords:** RhoA, Cdc42, FRET, local stimulation, Semaphorin-3A, GC retraction, NG108-15 cell line

## Abstract

Small RhoGTPases, such as Cdc42 and RhoA, are key players in integrating external cues and intracellular signaling pathways that regulate growth cone (GC) motility. Indeed, Cdc42 is involved in actin polymerization and filopodia formation, whereas RhoA induces GC collapse and neurite retraction through actomyosin contraction. In this study we employed Förster Resonance Energy Transfer (FRET) microscopy to study the spatio-temporal dynamics of Cdc42 and RhoA in GCs in response to local Semaphorin-3A (Sema3A) stimulation obtained with lipid vesicles filled with Sema3A and positioned near the selected GC using optical tweezers. We found that Cdc42 and RhoA were activated at the leading edge of NG108-15 neuroblastoma cells during spontaneous cycles of protrusion and retraction, respectively. The release of Sema3A brought to a progressive activation of RhoA within 30 s from the stimulus in the central region of the GC that collapsed and retracted. In contrast, the same stimulation evoked waves of Cdc42 activation propagating away from the stimulated region. A more localized stimulation obtained with Sema3A coated beads placed on the GC, led to Cdc42 active waves that propagated in a retrograde manner with a mean period of 70 s, and followed by GC retraction. Therefore, Sema3A activates both Cdc42 and RhoA with a complex and different spatial-temporal dynamics.

## Introduction

Axon outgrowth and guidance depend on the ability of growth cones (GCs) to detect molecular guidance cues in their extracellular environment and to respond with a dynamic remodeling of the cytoskeleton. It is well-established that repulsive GCs turning arises from the disruption and loss of F-actin superstructures and actomyosin contraction, while attractive GC turning entails asymmetrical incorporation of actin on the side of the GC close to the chemoattractant (Dent et al., 2011). The Rho protein family members of small GTPases act as molecular switches to control signal transduction pathways by cycling between a GDP-bound, inactive form, and a GTP-bound, active form (Raftopoulou and Hall, 2004). In their active state, they have a key role in the cytoskeleton reorganization as they act downstream of most guidance cue receptors (Hall, 1998; Luo, 2000; Dickson, 2001; Jaffe and Hall, 2005). In neurons, the Rho family members RhoA and Cdc42 regulate GC extension and axon outgrowth: RhoA triggers actin cytoskeleton rearrangement to support GC collapse and neurite retraction (Thies and Davenport, 2003; Wu et al., 2005), whereas Cdc42 promotes neurite outgrowth and GC filopodia formation (Ahmed et al., 2006; Hall and Lalli, 2010). However, the activation of the RhoA pathway has growth-promoting effects in cortical and hippocampal cell axons (Ahnert-Hilger et al., 2004; Ohnami et al., 2008).

The secreted guidance cue is Semaphorin-3A (Sema3A), a repulsive guidance molecule that generally causes GC collapse in neurons through microtubule and actin reorganization (Fan and Raper, 1995; Goshima et al., 2002; Tran et al., 2007; Zhou et al., 2008), helping steer both axons and migrating cells along the correct trajectory. Sema3A is thought to act as a dimer (Wong et al., 1999) binding to the complex of receptors neuropilin1/plexinA1, to initiate signal transduction pathways (Adams et al., 1997; Takahashi et al., 1997). Intracellular signaling of Sema3A has been extensively studied, also quite recently (Jongbloets and Pasterkamp, 2014; Worzfeld and Offermanns, 2014). In DRG neurons, Sema3A stimulation induces GC collapse of sensory neurons through RhoA effector ROCK and intra-axonal translation of RhoA mRNA, which, through an axonal 3′UTR targeting element, is localized in developing axons and GCs (Wu et al., 2005; Hengst et al., 2006). This local translation is a condition necessary and sufficient to allow Sema3A-mediated GC collapse. In contrast, the role of Cdc42 in Sema3A collapse response is still unclear with reported contradictory results (Jin and Strittmatter, 1997; Kuhn et al., 1999).

Bath application of Sema3A has several drawbacks: the entire neuron is exposed to Sema3A and the response could involve both the entire neuron and the GC (Brown et al., 2009). In the present study, we combined the sensitized emission Förster Resonance Energy Transfer (FRET) technique with local stimulation to observe the spatial and temporal activation of RhoA and Cdc42 following localized stimulation with Sema3A. We used two types of vectors for local delivery of molecules: microbeads and micro-sized lipid vesicles. Silica microbeads have been covalently functionalized on their surface with Sema3A and placed on the GC of interest with a sub-micrometric precision, allowing a more localized stimulation for 30 s. Thus, Sema3A beads were able to induce a localized response to Sema3A, although the guidance molecule was not in its native form. We then used lipid vesicles encapsulating Sema3A that were trapped with optical tweezers in front of the exploring GC and broken with short UV laser pulse. In this way we studied the delivery of Sema3A in its native form but with a less precise spatial localization of its delivery.

In this paper, we examine the role of RhoA and Cdc42 in NG108-15 neuroblastoma cells and we show that they are both involved in Sema3A morphological changes with specific spatio-temporal dynamics; we also show that Cdc42 (but not RhoA) exhibits a complex wave behavior. Although the existence of different models that prove both a crosstalk and a negative feedback between the Rho GTPases (Holmes et al., 2012; Marèe et al., 2012) is confirmed, our data support a more dynamic pattern for Cdc42 that reflects a higher degree of complexity for the Rho GTPase signaling.

## Materials and methods

### Cell cultures and transfection

NG108-15 neuroblastoma cells were purchased from Sigma Aldrich and grown in Dulbecco's modified Eagle's medium (DMEM) with 10% (v/v) fetal bovine serum (FBS) (Invitrogen), 100 μg/ml streptomycin and 100 units/ml in penicillin in a 5% CO_2_atmosphere at 37°C. For live cell imaging studies, cells were seeded on glass coverslips, coated for 3 h with laminin (50 μg/ml, L2020 Sigma) in 12-multiwell plates. Cells were transfected 24 h later with intermolecular RhoA/Cdc42 FRET sensors (Murakoshi et al., 2011) using Metafectene reagent (Biontex Laboratories) following the manufacturer's protocol and imaged 1 day after transfection. mEGFP-RhoA-C1 (Addgene plasmid #29674), mEGFP-Cdc42-C1 (Addgene plasmid #29673), mCherry-Rhotekin(8–89)-mCherry-C1 (Addgene plasmid #29675) and mCherry-Pak3(60–113)/S74A/F84A-mCherry-C1 (Addgene plasmid #29676) were a gift from Ryohei Yasuda (Murakoshi et al., 2011).

### Immunofluorescence

All these steps were performed at room temperature (20–22°C) and coverslips were rinsed with phosphate-buffered saline (PBS) between each step. Cells were fixed in freshly prepared 4% paraformaldehyde containing 0.15% picric acid in PBS, permeabilized in 0.1% Triton X-100 for 10 min and blocked with 0.5% BSA (all from Sigma-Aldrich, St.Louis, MO) in PBS for 1 h. Cells were then incubated with primary goat polyclonal antibody against neuropilin1 (Santa Cruz Biotechnology, Santa Cruz, CA) and rabbit polyclonal antibody against plexinA1 (Santa Cruz Biotechnology, Santa Cruz, CA) for 1 h. The secondary antibodies were donkey anti-rabbit 488 and donkey anti-goat 594 Alexa (Invitrogen, Life Technologies, Gaithersburg, MD, USA) and the incubation time was 30 min. Nuclei were stained with 2 μg/ml Hoechst 33342 (Sigma-Aldrich, St.Louis, MO) in PBS for 5 min. Coverslips were inverted and mounted on the glass side using Vectashield Mounting Medium and were then examined on a Leica DM6000 microscope (Leica Microsystems GmbH, Germany) using a 100 × magnification and 1.42 NA oil-immersion objective. Fluorescent images were processed using Leica LCS Lite and Image J by W. Rasband (developed at the U.S. National Institutes of Health and available at http://rsbweb.nih.gov/ij/). For the colocalization analysis, each image was captured applying the same exposure and gain settings per channel and using Volocity 5.4 3D imaging software (PerkinElmer, Coventry, UK).

### Vesicle preparation

Detailed experimental procedures are described in our companion papers (Pinato et al., 2011, 2012). Single vesicles, with a diameter of 1–5 μm, were obtained with the lipid film hydration method using a hydration solution containing BSA or Sema3A, and were then identified, trapped and positioned at the location of interest (Ichikawa and Yoshikawa, 2001).

### Beads functionalization and immunohistochemistry

1.5 μm large silica beads coated with COOH groups (Kisker-biotech, cat. PSi-1.5COOH) were functionalized using PolyLink Protein Coupling Kit (Bangs Laboratories Inc., cat. PL01N), following the manufacturers protocol. Briefly, about 1.4 × 10^5^ beads were incubated with 500 ng of Sema3A, in the presence of 20 μg/μL EDAC for 1 h, at room temperature and stored in Storage Buffer at 4°C.

Coated and un-coated microspheres were washed in PBS and incubated for 1 h, at room temperature, with anti-Sema3A (1:50, Sigma). Beads were then centrifuged, washed and incubated for 30 min, at room temperature, with donkey anti-goat Alexa 594 (Invitrogen) and finally analyzed using Fluorescence Microscopy (Nikon Eclipse TE-2000-E).

### Optical manipulation and FRET microscopy setup

**Figure 1:**
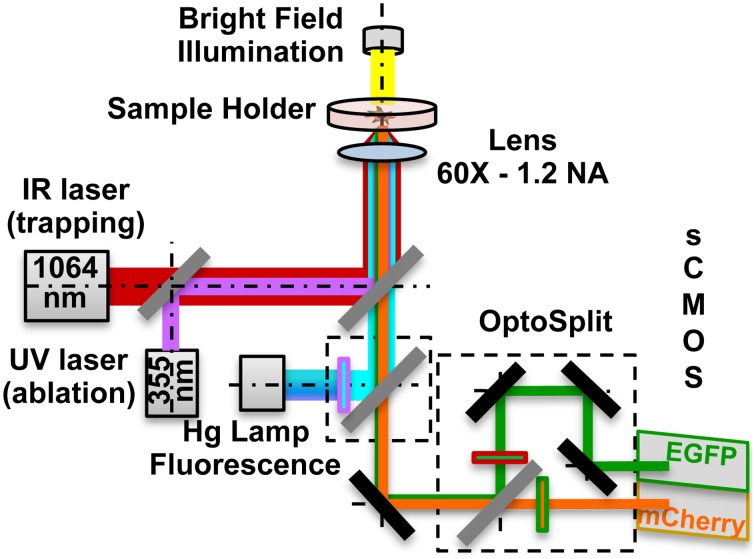
**Optical manipulation and FRET imaging setup**. The IR laser beam (1064 nm) is aligned to the UV laser micro-dissection beam (355 nm) and then both are directed into the pupil of the microscope objective (60X - 1.2 NA). The sample is illuminated both by a white light source and a Hg fluorescence lamp. The light coming from the Donor and the Acceptor fluorophores in the sample is separated by a dual emission image splitter and the two images are formed on the two halves of the sCMOS camera sensor.

Local delivery was achieved by optical trapping and manipulation of single vesicles at a specific position nearby the cells, followed by vesicles photolysis, to release active molecules. To increase the localization of the stimulus, single beads were optically trapped and maintained in contact with the GCs for 30 s. RhoGTPases activation was monitored by FRET microscopy. The setup, depicted in Figure 1, was developed around an inverted microscope (Nikon Eclipse TE-2000-E) adding three modules: (1) Infrared (IR) laser tweezers (custom built); (2) Ultraviolet (UV) laser micro-dissection system (MMI- CellCut Plus, MMI, Zurich, Switzerland); (3) Image splitter (OptoSplit II LS, Cairn Research, UK).

The first two modules are described in detail in our companion papers (Pinato et al., 2011, 2012). The trapping laser beam, generated by a 1064 nm fiber laser, is aligned with the micro-dissection UV beam and then focused on the sample through the same lens (Nikon, 60X water immersion, numerical aperture NA-1.2). The fluorescence path includes a 100 W Hg Lamp, excitation filters for EGFP (445/30 nm, Chroma, USA) and mCherry (550/30 nm). Light emitted by the sample is directed to the image splitter, where a dichroic mirror (585 nm) splits the light into Donor (EGFP) and Acceptor (mCherry) channels. After passing through the emission filters (EGFP: 515/30 nm; mCherry: 625/30 nm) each of the two beams is imaged on one of the two halves of a sCMOS sensor (Orca Flash 4.0, Hamamatsu), thus allowing the acquisition of the donor and of FRET emissions simultaneously. All live cells and FRET microscopy experiments were performed on the setup previously described. Imaging was performed in a Ringer's Solution and during the experiments cells were kept at 37°C, using a temperature controller (TempControl 37-2, PeCon, Germany). Experiments with the same coverslip always lasted less than 60 min. For bath applied stimulation assays time-lapse DIC images were acquired every 500 ms with a Nikon, 20X (NA: 0.25) objective in case of population studies, and with a Nikon, 60X (NA: 1.2) objective in case of single cell studies, from 90 s before adding the Sema3A to 20 min after it. For local stimulation assays, we trapped the lipid vesicle or the bead with the optical tweezers and we positioned it in front of, or in contact with, the selected GC. We then acquired simultaneously the Donor and Acceptor (FRET) channels for 15 min after the vesicle photolysis. All acquisitions were done with the sCMOS sensor at 16-bit depth. The exposure time for the FRET channels acquisition was 1 s at binning 4 × 4.

### Ratiometric corrections and image analysis

Ratiometric corrections and ratio calculations to generate activity images were performed using the Biosensors 2.1 MATLAB package (Danuser laboratory: http://lccb.hms.harvard.edu/software.html). All images were sequentially shading-corrected and background-subtracted; an optional photobleaching correction was also applied. Binary masks, with values equal to 1 inside the cells and 0 elsewhere, were extracted by thresholding the EGFP image (since it presented the highest signal-to-noise ratio). Control cells expressing either EGFP alone or mCherry alone were used to obtain bleedthrough coefficients necessary for the correction of the FRET image. The final ratio image, obtained dividing the completely corrected FRET image by the EGFP image, was used as a measure for the RhoA and Cdc42 activity.

Further image analysis was performed by ImageJ and custom-built codes were written in Matlab (Mathworks). To describe RhoA and Cdc42 activation, rectangular areas (size 10 × 20 μm) were generated selecting regions of interest in the normal direction with respect to the edge dynamics of the GCs; montage images were then composed by taking one image every 60 or 120 s for the entire duration of the experiment. The quantification of the FRET ratio was performed in stalling, protruding and retracting regions of the cell edge, and the results were normalized by the mean intensity of the ratio in the stalling regions. To measure the temporal dynamics of RhoA and Cdc42, a square area of 4 μm^2^ was selected in the region of interest previously considered for image montages, so that no movement of the edge could interfere with the measurements; the intensity was calculated for every time point and then the measurements were normalized using the following equation: $\Delta F=\frac{{F-F}_{0}}{F_{0}}$, where *F* is the intensity at the time point *t* and *F*_0_ is the intensity at the first frame. Cell edges were detected for each frame by intensity-thresholding of the Ratio final image and edge dynamics were calculated along the central line of the rectangles, previously considered for the image montages. Edge and RhoGTPases activation dynamics were then plotted together in a graph, as a function of time. To measure the period of the waves in the different cases, one or more areas were selected in the retracting GCs, as previously described for temporal dynamics measurements, and then the wave period was calculated extrapolating the time between two maxima from the intensity vs. time plots.

### Statistical analysis

All quantitative results have been obtained from at least three independent experiments and expressed as the mean ± SEM. Experimental data were analyzed by Student's *t*-test and One-Way analysis of variance (ANOVA). Differences among samples were considered statistically significant when *p* < 0.05.

## Results

### The spontaneous dynamics of RhoA and Cdc42 in NG108-15 cell line

NG108-15 neuroblastoma cells are a good model system for neuronal signaling and growth (Smalheiser, 1991a,b; Goshima et al., 1993; Tsuji et al., 2011). In the absence of adherent or diffusive signaling gradients, NG108-15 cells exhibit both a complete collapse of the GC followed by a full or partial retraction of the neurite and the transient collapse of the GC structures (Rauch et al., 2013). Therefore, to measure the spontaneous activity of RhoA and Ccd42 in GCs, we transiently overexpressed in NG108-15 cells the intermolecular RhoA/Cdc42 probes based on FRET (Murakoshi et al., 2011). These probes consist of two components: wild type RhoA/Cdc42 tagged with monomeric enhanced green fluorescent protein (mEGFP) and their binding partner, Rho GTPase binding domain (RBD) of Rhotekin/Pak3, doubly tagged with mCherry (mCherry-RBD-mCherry) (Murakoshi et al., 2011). The expression of the intermolecular RhoA/Cdc42 probes was carefully titrated to levels that did not alter the normal neurite outgrowth. GFP, mCherry and FRET images were captured 18-20 h post-transfection using a Dual-view image splitting device. After background and spectral bleed-through correction, the GFP/mCherry ratio was measured and correlated with the activation levels of RhoA and Cdc42 during spontaneous protrusion and retraction events.

NG108-15 neuroblastoma cell line shows neurite outgrowth when plated on glass slides that have been coated with laminin. To examine the effect of RhoA and Cdc42 transfection on NG108-15 cells, we compared the morphologies of cells transfected with either RhoA or Cdc42 (Figures S1A,B). Transfection of Cdc42 induced significant neurite outgrowth compared with RhoA transfected cells that appeared more flatted (Figures S1A–D). Furthermore, most of the Cdc42-transfected cells were polarized with long neurite-like processes that were generally branched and exhibited micro-spikes along their length (Figures S1B–D).

**Figure 2:**
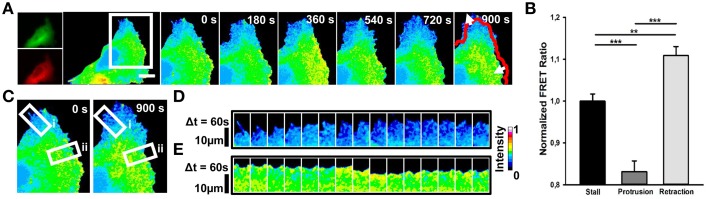
**Spontaneous RhoA dynamics in NG108-15 cells**. **(A)** Ratiometric FRET live imaging of a representative RhoA-FRET neuroblastoma cell (n = six experiments). Consecutive frames were taken every second (left: generation of a ratiometric image) for 15 min. Insets on the right show a time series (1 frame every 3 min) of the magnified region denoted by the white box in the left image. Arrows indicate protrusion and retraction directions. The red line shows the initial edge profile. Scale Bar: 20 μm. **(B)** Quantification of mean RhoA activity (FRET ratio) in 50 stalling, protruding and retracting sections along the cell edge from RhoA neuroblastoma cells from two experiments. ^**^p < 0.01, ^***^p < 0.001 using ANOVA test (**C**–**E**) Montage images showing the dynamics of RhoA activation in protruding **(D)** and retracting sections **(E)** along the cell edge. **(C)** Images showing the regions of interest selected for the montage (white boxes) (i, protruding region; ii, retracting region). **(D,E)** Montage images highlighting a low RhoA activity in protruding regions **(D)** and a higher RhoA activity in retracting ones **(E)**. Intensity scale on the right in **(D)** applies to **(A,C–E)**.

In RhoA transfected cells, we observed that elevated RhoA activity (Figures 2A,B; mean FRET ratio: 1.109) correlates with edge retraction, whereas reduced RhoA activity (Figures 2A,B; mean FRET ratio: 0.831) leads to edge protrusion. To investigate how spontaneous RhoA activity relates to edge dynamics, we scanned RhoA activity in both protrusion (Figures 2C,D) and retraction regions (Figures 2C,E) of one representative cell at 60 s intervals. This confirmed that edge retraction coincides with a boost in RhoA activity compared with a protrusion event.

**Figure 3:**
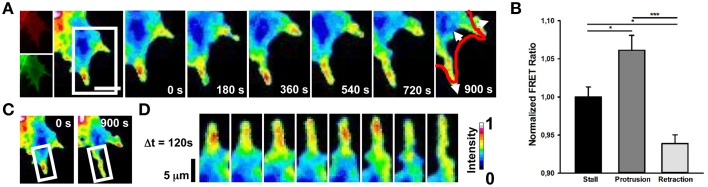
**Spontaneous Cdc42 dynamics in NG108-15 cells**. **(A)** Ratiometric FRET live imaging of a representative Cdc42-FRET neuroblastoma cell (n = six experiments). Consecutive frames were taken every second (left: generation of a ratiometric image) for 15 min. Insets on the right show a time series (1 frame every 3 min) of the magnified region denoted by the white box in the left image. Arrows indicate protrusion and retraction directions. The red line shows the initial GC profile. Scale Bar: 20 μm. **(B)** Quantification of mean Cdc42 activity (FRET ratio) in 30 stalling, protruding and retracting sections along the cell edge from Cdc42 neuroblastoma cells from two experiments. ^*^p < 0.05, ^***^p < 0.001 using ANOVA test **(C,D)** Montage images showing the dynamics of Cdc42 activation in a protruding region of the cell. **(C)** Images showing the region of interest selected for the montage (white boxes). **(D)** Montage images highlighting hotspots of Cdc42 in the protruding region. Intensity scale on the right in **(D)** applies to **(A,C,D)**.

Cdc42 activity has been implicated in the regulation of filopodia and lamellipodia extension in many cell types (Nobes and Hall, 1995; Peng et al., 2003; Nalbant et al., 2004; Myers et al., 2012). We then imaged Cdc42 transfected cells and we found that membrane-ruffling and filopodia extension strongly correlate with hot spots of Cdc42 activity (Figure 3B; mean FRET ratio of 1.061) with respect to cell edges without protrusions (Figure 3B; mean FRET ratio of 0.938) (Figure 3). Therefore, these findings indicate that a selective activation of RhoA and Cdc42 correlates spatially and temporally with retraction and extension events in NG108-15 cell line.

### Sema3A induces GC retraction in NG108-15 cell line

In order to study the NG108-15 behavior after Sema3A stimulation, we first observed the distribution of Sema3A receptors neuropilin1 and plexinA1 using immunofluorescence microscopy after double staining with antibodies against neuripilin1 and plexinA1: both the Sema3A receptors were found in GCs of NG108-15 cell line (Figure S1E). This observation was consistent with the distribution of neuropilin1 and plexinA1 shown for hippocampal neurons (Pinato et al., 2012). Since bath application of Sema3A could have several drawbacks (Brown et al., 2009), we examined the effect of local stimulation with Sema3A filled lipid vesicles on GC in NG108-15 cells. We had previously demonstrated that local stimulation with vesicles encapsulating guidance molecules can allow a controlled spatiotemporal stimulation of hippocampal neurons that mimics better the natural behavior of the guidance molecules themselves (Pinato et al., 2012).

**Figure 4:**
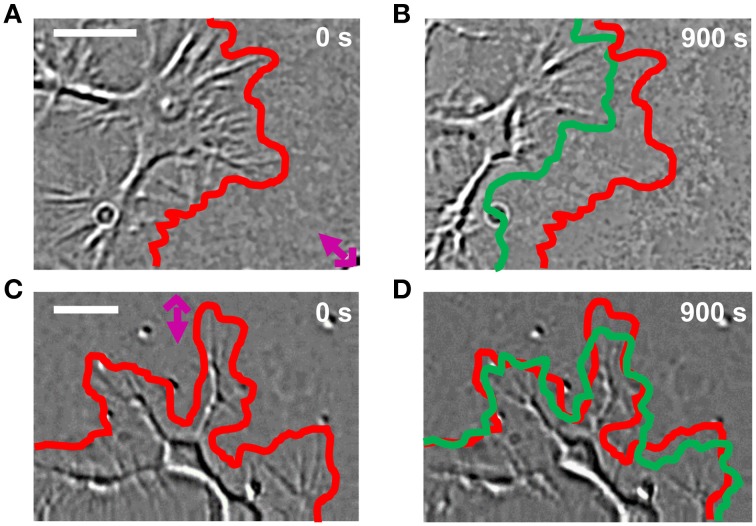
**GC behavior of NG108-15 cells stimulated with Sema3A**. **(A,B)** DIC images of a representative cell locally stimulated with lipid vesicles encapsulating 1 μM of Sema3A at 0 **(A)** and 900 s **(B)** from the delivery. The red and green lines represent respectively the initial and final GC profile. The pink cross with the arrow represents the position of the liposome. **(C,D)** The same as in **(A,B)**, but the representative cell is locally stimulated with lipid vesicles encapsulating 1 μM of BSA. Frames were taken every second for 15 min in both cases. Contrast and brightness enhancement were applied to all images, followed by bandpass filtering of the spatial frequencies to enhance the edge contrast.

Lipid vesicles, with a diameter varying between 1 and 5 μm, were encapsulated with 10^3^–10^4^ molecules of Sema3A (Pinato et al., 2011, 2012). Local stimulation with lipid vesicles encapsulating Sema3A led to a rapid collapse and retraction of the GC (Figures 4A,C): retraction was already detectable at 100 s after stimulation with Sema3A and reached a plateau between 5 and 6 min. After local stimulation with BSA, no comparable retraction was observed (Figures 4B,D).

### Local stimulation by Sema3A induces RhoA activation followed by the GC retraction

**Figure 5:**
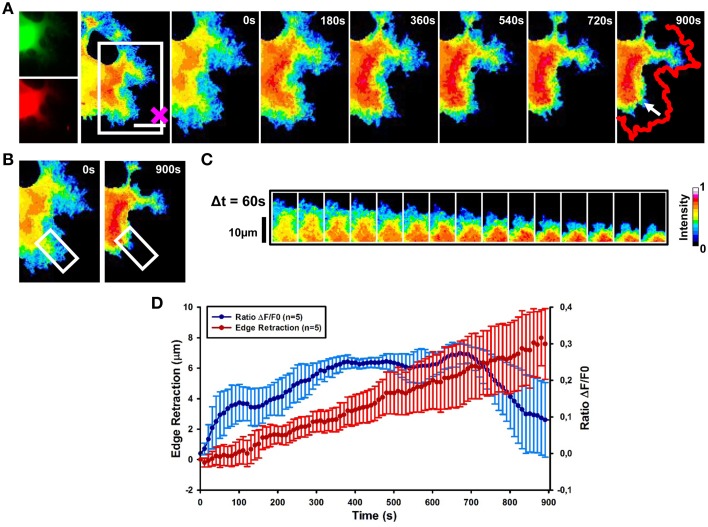
**RhoA activation dynamics upon local release of Sema3A**. **(A)** Ratiometric FRET live imaging of a representative RhoA-FRET neuroblastoma cell upon Sema3A stimulation. Frames were taken every second (left: generation of a ratiometric image) for 15 min (the cross indicates the position of the lipid vesicle filled with Sema3A). Insets on the right show a time series (1 frame every 3 min) of the magnified region denoted by the white box in the left image. Arrow indicates the retraction direction. The red line shows the initial edge profile. Scale Bar: 20 μm. **(B,C)** Montage images showing the dynamics of RhoA activation in the stimulated cell GCs. **(B)** Images showing the region of interest selected for the montage (white boxes). **(C)** Montage images highlighting a progressive increase of RhoA activation in the retracting GC. Images were taken at 60 s intervals. Intensity scale on the right in **(C)** applies to **(A–C)**. **(D)** Plot of average RhoA activity (Δ*F*/*F*_0_) from five experiments. RhoA dynamics was followed for 15 min after Sema3A stimulation. FRET ratio is represented as a blue line; edge retraction is defined as a red line. Data are shown as Mean ± SEM.

To investigate the spatio-temporal activation of RhoA after local stimulation with Sema3A, we combined FRET with optical tweezers set-up to induce the local delivery of vesicles encapsulating Sema3A (Figure 1-see Methods section). Local application of Sema3A leads to an increase of RhoA activity in the central region of the GC followed by GC collapse and retraction (Figure 5A and Supplementary Movie 1). Ratiometric FRET imaging highlighted that RhoA activity increases rapidly, within 30 s after Sema3A local stimulation, reaching the maximum value within 400 s (Figures 5B,C). A more detailed analysis of the time dependence of RhoA activity and GC edge retraction showed that the GC begins to retract within 100–120 s after Sema3A local stimulation, reaching a complete collapse and retraction within 15 min (Figure 5D; data from five experiments). The delay between the increase of RhoA activity and the GC retraction can be explained with the recruitment of RhoA binding proteins necessary for cytoskeletal rearrangement. When we stimulated RhoA transfected cells with vesicles encapsulating BSA (Figures S2A–C), no comparable retraction (Figure 2) was observed.

Thus, our results strongly suggest that Sema3A local stimulation leads to an activation of RhoA associated to GC collapse and retraction.

### Cdc42 activation exhibits a wave-like behavior

**Figure 6:**
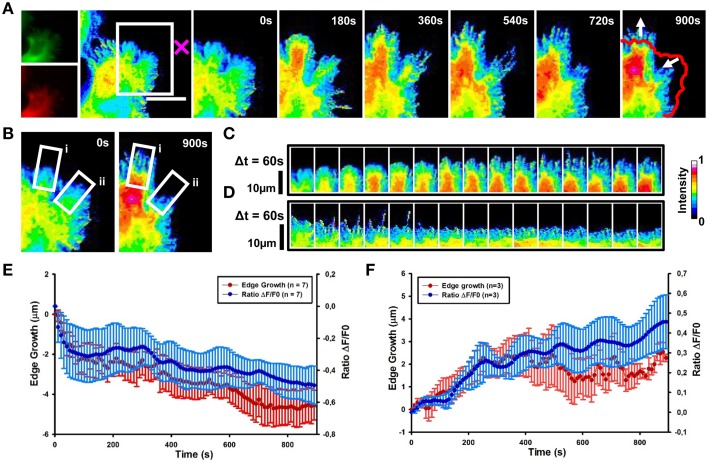
**Cdc42 dynamics upon local delivery of Sema3A**. **(A)** Cdc42 activity determined by ratiometric FRET live imaging. Frames were taken every second (left: generation of a ratiometric image) for 15 min after stimulation (the cross indicates the position of the lipid vesicle encapsulating Sema3A). Insets on the right show a time series (1 frame every 3 min) of the magnified region denoted by the white box in the left image. Arrows indicate retraction and protrusion directions. The red line shows the initial edge profile. Scale Bar: 20 μm. **(B–D)** Montage images showing the dynamics of Cdc42 activity in protruding and retracting regions of the cell. **(B)** Images showing the regions of interest selected for the montage (white boxes) (i, protruding region; ii, retracting region). **(C,D)** Montage images highlighting a decrease of Cdc42 activity in the retracting region of the cell facing the lipid vesicle filled with Sema3A **(C)** and an increase of Cdc42 activity in the protruding region, away from the vesicle **(D)**. Images are taken at 60 s intervals. Intensity scale on the right in **(D)** applies to **(A–D)**. **(E)** Plot of average Cdc42 activity (Δ*F*/*F*_0_) vs. edge growth from seven experiments. Negative values are defined as retraction. FRET ratio is represented as a blue line; edge growth is defined as a red line. **(F)** The same as in **(E)**, but in this case data are obtained from three experiments. Positive values are defined as edge protrusion. Data are shown as Mean ± SEM.

We then determined the spatial and temporal dynamics of activated Cdc42 in response to Sema3A local delivery (Supplementary Movie 2). Ratiometric FRET imaging showed that Cdc42 activity decreases within 60 s from Sema3A stimulation almost in synchrony with the cell edge retraction (Figures 6A,B,D,E and Figure S3; data from seven experiments).

**Figure 7:**
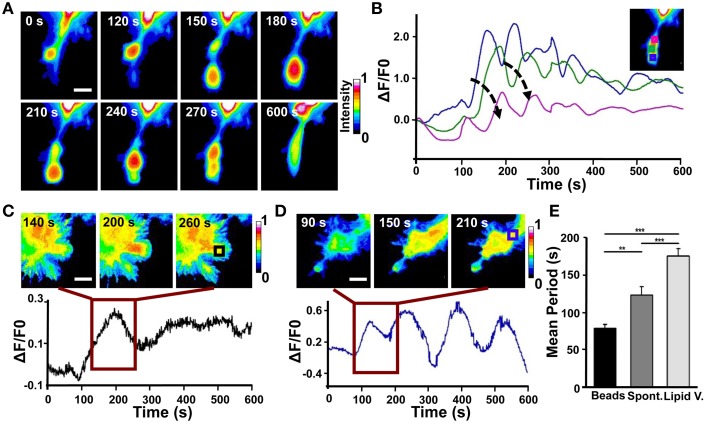
**Analysis of Cdc42 wave-like behavior**. **(A)** Time series of the active Cdc42 retrograde waves upon local stimulation with Sema3A-coated beads (Representative of three experiments). **(B)** Plot of average Cdc42 activity (Δ*F*/*F*_0_) in the regions defined by the colored squares in the inset image at the top right. The analyzed cell is the same shown in **(A)**. Arrows indicate the temporal progression of the oscillations. **(C)** Top: Time series (1 frame every 60 s) of the Cdc42 oscillatory behavior upon local stimulation with lipid vesicles filled with Sema3A for the time defined by the dark red square in the bottom plot; Bottom: Plot of average Cdc42 activity (Δ*F*/*F*_0_) in the region defined as a black square in the image at the top right. **(D)** Top: Time series (1 frame every 60 s) of the Cdc42 wave behavior during spontaneous motion for the time defined by the dark red square in the bottom plot. Bottom: Plot of average Cdc42 activity (Δ*F*/*F*_0_) in the region defined as a blue square in the image at the top right. **(E)** Quantification of the period of the oscillations both during spontaneous motion and upon local stimulation. Mean period ± SEM of 10 periods from 3 experiments for bead stimulation, 10 periods from 2 experiments for spontaneous motion and 10 periods from 3 experiments for lipid vesicles stimulation. ^**^p < 0.01, ^***^p < 0.01 using ANOVA test. Scalebars in **(A,C,D)**: 10 μm.

In 43% of cases, we observed that in the region far from the stimulus Cdc42 activity increases within 120 s after Sema3A stimulation followed by edge advancement, lamellipodia ruffling and filopodia extension (Figures 6A–C,F). Plotting Cdc42 activity against time showed waves of active Cdc42 between the front and back of the protruding region, with a mean period of 155 ± 9 s (Figures 7C,E; data from three experiments). The delay between Cdc42 activation and Sema3A stimulation indicates that the protrusion event in the region far from the local release of Sema3A is a reaction to the repulsive stimulus. Consistently, BSA local stimulation seemed not to affect the activity of Cdc42 in transfected cells (Figures S2D–F).

In order to better characterize Cdc42 activity in response to Sema3A local stimulation, we used 1.5 μm microbeads coated with Sema3A that allow a more localized stimulation (D'Este et al., 2011) (Figure S4). Live FRET imaging of GCs transfected with Cdc42 and stimulated with Sema3A-beads showed the appearance of recurring waves of active Cdc42 almost in synchrony with the retraction of the GC (Figure 7A). Plotting Cdc42 intensity against time from three different areas of the stimulated GC revealed a burst of Cdc42 activity within 100–120 s from the local stimulation and highlighted the propagation of Cdc42 traveling waves from the front to the back of the retracting GC (Figure 7B). In spontaneous GC collapse and retraction we observed again Cdc42 active waves, but with a different periodicity (Beads: 70 ± 5 s vs. Spontaneous: 110 ± 10 s) (Figures 7D,E). Indeed, we found that Cdc42 active waves have different periods in all three cases described herein (Lipid vesicles, Spontaneous, and Coated Beads) reflecting the different cellular behavior in response to the stimulus applied (Holmes et al., 2012; Marèe et al., 2012).

Taken together, these findings show that (a) Cdc42 has a traveling wave behavior with a different periodicity, highlighting its complex spatio-temporal dynamic; (b) that, in all the cases observed, a variation of Cdc42 activity upon Sema3A stimulation is accomplished with a leading edge retraction in the region of the stimulus; (c) that, in few cases, Sema3A local release induces the formation of active Cdc42 waves away from Sema3A stimulation that produced new lamellipodia and filopodia.

## Discussion

By combining FRET with local stimulation we have demonstrated that local delivery of Sema3A induces an activation of RhoA followed by a rapid GC retraction. The use of two different approaches (beads and liposome vesicles) for Sema3A local stimulation of Cdc42 transfected cells, both based on optical manipulation, led to a better characterization of Cdc42 wave behavior. Sema3A release from the vesicles induced a decrease in Cdc42 activity followed by an edge retraction. In regions distant from the stimulus, active waves of Cdc42 resulted in a marked increase in cell dynamic with lamellipodia-ruffling and filopodia extension. Using 1.5-μm diameter Sema3A-immobilized microbeads, we observed again Cdc42 active waves that here resulted in GC retraction. In previous papers (Pinato et al., 2011, 2012), the local delivery was used to estimate the minimum number of Sema3A and Netrin1 molecules necessary to induce retraction and growth, respectively. The present study expands previous observations by combining for the first time local stimulation with the FRET technique underlying a fine spatial and temporal regulation of RhoGTPase activities after Sema3A stimulus.

### Rhoa and Cdc42 have a different spontaneous activity in NG108-15 neuroblastoma cells

Rho GTPases are key molecular switches that affect multiple cellular functions including cell migration and polarity, vesicle trafficking, cytokinesis and endocytosis (Etienne-Manneville and Hall, 2002; Govek et al., 2005; Boureux et al., 2007; Haesman and Ridley, 2008; Pertz, 2010). They are important players in the transmission and integration of signals that control the cytoskeleton: in response to extracellular signals, they induce changes in the organization of actin cytoskeleton to allow different biological processes like morphogenesis, chemotaxis, axonal guidance, and cell cycle progression (Ridley and Hall, 1992; Gallo and Letourneau, 2004; Sit and Manser, 2011; Gomez and Letourneau, 2014). In N1E-115 neuroblastoma cell line, Cdc42 promotes the formation of filopodia and lamellipodia, whereas RhoA causes GC collapse and neurite retraction (Kozma et al., 1997; van Leeuwen et al., 1997; Sarner et al., 1997). Consistent with this view, on a laminin substrate, NG108-15 cells expressing Cdc42 exhibited long processes with rather simple GCs and branched neurites with several actin microspikes along their length. On the contrary, expression of RhoA often resulted in cell rounding and flattening without the appearance of stress fibers or focal contact (Figure S1). Spatial and temporal monitoring of spontaneous activity of RhoA and Cdc42 in NG108-15 cells allowed us to directly compare cell motility with levels of active RhoA and Cdc42. We found that filopodia and lamellipodia protrusions correlate with Cdc42 activation in these subcellular domains (Figure 3), whereas RhoA activity increases and decreases in synchrony with cell leading edge retraction and protrusion (Figure 2).

### Local release of Sema3A induces a rapid GC collapse and retraction

The role of Rho-family GTPases in response to several guidance cue stimulations has been extensively studied in order to address their role in F-actin dynamics and organization that determine axon guidance, GC behavior and axon extension (Gallo and Letourneau, 2004). Attractive cues such as brain-derived neurotrophic factor (BDNF) activate Cdc42 and Rac (Yuan et al., 2003; Cheung et al., 2007; Myers et al., 2012; Tep et al., 2012), whereas repulsive cues like Slit or Eph/ephrin have been shown to induce a reduction of Cdc42 activity at GC periphery and an activation of RhoA/ROCK pathway respectively (Wahl et al., 2000; Myers et al., 2012; Takeuchi et al., 2015). However, it is well-established that small GTPases are important components of Semaphorin (Sema)/plexin axon guidance signaling (Kruger et al., 2005; Tran et al., 2007). The Sema family of secreted, transmembrane and GPI-linked proteins is one of the largest families of axon guidance cues and guides the growing axons by repelling them or preventing them from entering certain regions (Yazdani and Terman, 2006). The prototypic member of this family is the diffusible repulsive guidance cue Sema3A that induces collapse of GCs through the interaction with its receptor plexinA1 (Fan and Raper, 1995; Nakamura et al., 2000; Brown and Bridgman, 2009). In our experiments, Sema3A local delivery leads to a rapid GC collapse and retraction in NG108-15 neuroblastoma cell line (Figure 4) in agreement with similar experiments in DRG neurons (Brown et al., 2009).

### Sema3A local stimulation activates RhoA and Cdc42 with specific spatio-temporal dynamics

Previous findings have indicated an inhibitory role for RhoA in inducing neurite extension (Kozma et al., 1997; Dickson, 2001; Wu et al., 2005). Consistently, we found that Sema3A local release leads to RhoA activation within 30 s, causing a delayed GC retraction (100–120 s from stimulation) with a correlation between the levels of RhoA activity and Sema3A-induced morphological changes (Figure 5). This delay suggests that GC collapse and retraction could require RhoA recruitment of its binding partners.

In Cdc42 transfected cells, local delivery of Sema3A caused a decrease of Cdc42 activity within 60 s from the stimulation (Figure 6). Cdc42 showed a wave behavior with a retrograde flow that proceeded almost in synchrony with cell retraction toward the repulsive cue (Figure 6). In a few cases, Sema3A local release induced the formation of active Cdc42 propagating waves away from Sema3A stimulation that generated new lamellipodia and filopodia suggesting a role for Cdc42 as an important component of actin *t*-waves (Lim et al., 2008; Flynn et al., 2009; Mori et al., 2011; Allard and Mogilner, 2013) (Figure 6).

Local stimulation with Sema3A coated beads induced the formation of active Cdc42 waves that propagated from the edge of the GC to the center of the cell with a period of 70 s (Figure 7). The same active Cdc42 waves were found in spontaneous GC collapse and retraction in response to repellent signals naturally occurring in neuronal cell culture, but with a mean period of 110 s (Rauch et al., 2013). How differently Sema3A stimulation can influence Cdc42 wave behavior is still an open question. One possibility is that these different Cdc42 dynamic wave patterns reflect the heterogeneity of the cell population or differential activity states of the cell. Alternatively, bead mechanical stimulation could induce calcium oscillations within the cell that influence actin *t*-waves through the modulation of Cdc42 activity (Wu et al., 2013). Although previous models have highlighted a fine spatio-temporal crosstalk between Rho GTPases that exhibit sustained polarization by a wave-pinning mechanism (Mori et al., 2008; Holmes et al., 2012; Marèe et al., 2012), our data show a wave-propagation mechanism for Cdc42 but not for RhoA and provide clear evidence for a higher degree of complexity in Rho GTPase signaling network (Pertz, 2010).

Our findings clearly indicate that the combination of the FRET technique with local stimulation provides new tools for the study of cytoskeleton rearrangements in response to guidance cue stimulations, highlighting the dynamic spatial and temporal regulation of Rho GTPases.

### Conflict of interest statement

The authors declare that the research was conducted in the absence of any commercial or financial relationships that could be construed as a potential conflict of interest.
