# Supplementary material for: Cdc42 and RhoA reveal different spatio-temporal dynamics upon local stimulation with Semaphorin-3A
Source: Front Cell Neurosci. 2015 Aug 26;9:333. doi: 10.3389/fncel.2015.00333 (PMC4549648; doi:10.3389/fncel.2015.00333)
Supplement: Supplementary file 3 [file DataSheet1.PDF]

Supplementary Information:

Cdc42 and RhoA reveal different spatio-temporal dynamics upon local stimulation with Semaphorin-3A

Federico Iseppon[1], Luisa M.R. Napolitano[1,2], Vincent Torre[1,*] and Dan Cojoc[3,*]

[1]Neurobiology Sector, International School for Advanced Studies (SISSA), Trieste, Italy.
[2]Structural Biology Laboratory, Elettra-Sincrotrone Trieste S.C.p.A., Area Science Park, Basovizza, Trieste, Italy.
[3]Institute of Materials – National Research Council (IOM-CNR), Area Science Park-Basovizza, Italy

***Correspondence:** Vincent Torre, Neurobiology Sector, International School for Advanced Studies (SISSA), via Bonomea 265, Trieste 34136, Italy. E-mail: torre@sissa.it.
Dan Cojoc, Institute of Materials – National Research Council (IOM-CNR), Area Science Park- Basovizza, Trieste 34149, Italy. E-mail: cojoc@iom.cnr.it

Keywords: RhoA, Cdc42, FRET, local stimulation, Semaphorin3A, growth cone retraction, NG108-15 cell line

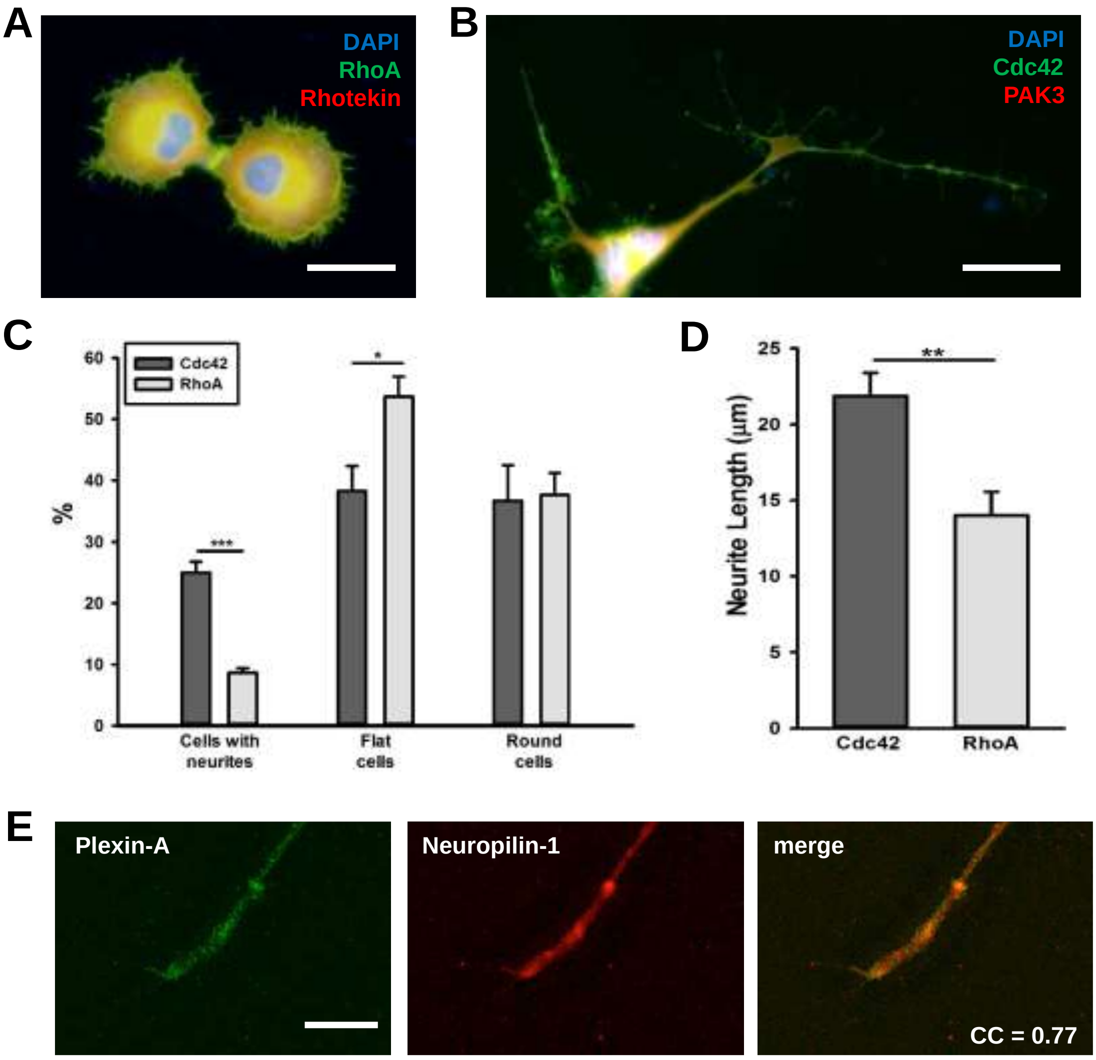

Figure S1: Cdc42 and RhoA expression in NG108-15 cell line. NG108-15 neuroblastoma cells were plated on laminin and transfected with either RhoA/Rhotekin **(A)** or Cdc42/Pak3 **(B)**. The values in **(C)** and **(D)** represent the percentage of transfected cells that exhibit neurite (C) and the neurite lenght (D) of 500 cells from four experiments. *p<0.05; **p<0.01; ***p<0.001 using ANOVA test **(E)** Immunostaining of NG108-15 growth cones with antibodies for plexinA1 (green) and neuropilin1 (red). As shown in the merged image, NG108-15 cells express both subunits needed to form a functional Sema3A receptor widely in the growth cones. Scale Bar: 5µm. Co-localization percentage: 77%.

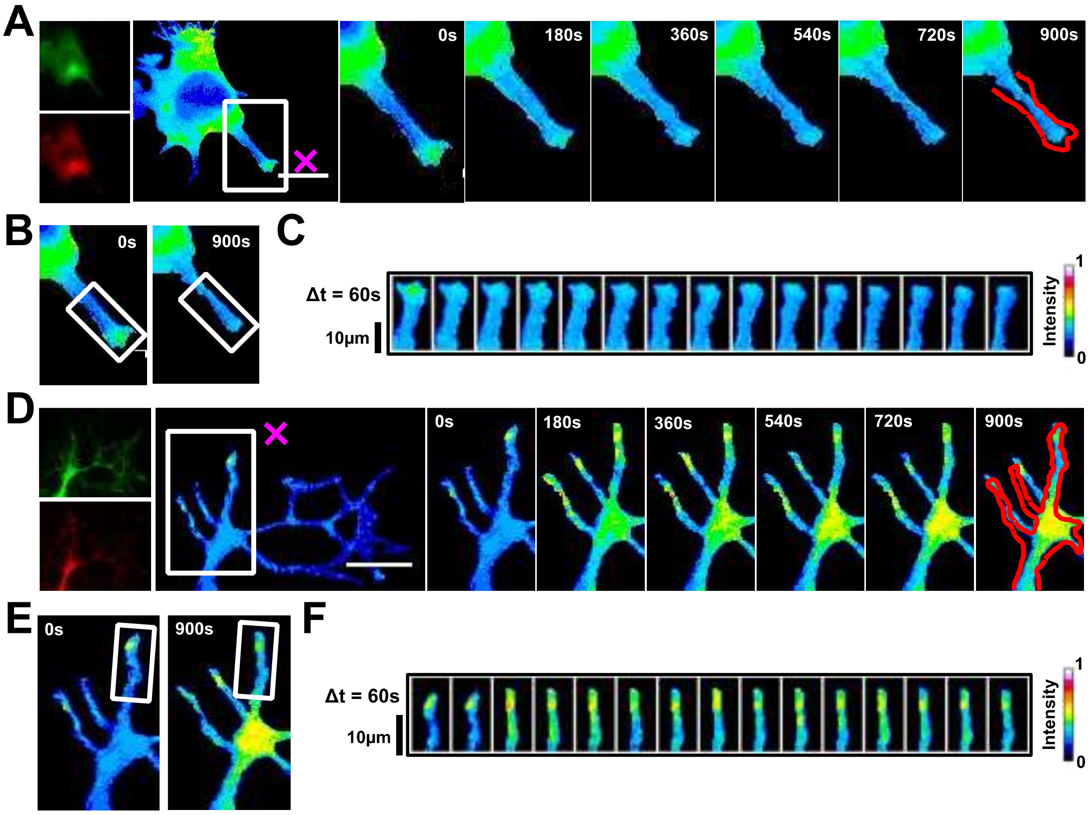

Figure S2: RhoA and Cdc42 dynamics in NG108-15 cells upon local stimulation with BSA-filled liposome. (A) RhoA activity determined by ratiometric FRET live imaging. Frames were taken every second (left: generation of a ratiometric image) for 15 minutes after the stimulation (the cross indicates the position of the lipid vesicle encapsulating BSA). Insets on the right show a time serie (1 frame every 3 minutes) of the magnified region denoted by the white box in the left image. The red line shows the initial edge profile. Scale Bar: 20µm. **(B** and **C)** Montage images showing RhoA dynamics in the stimulated cell growth cones. (B) Images showing the region of interest selected for the montage (white boxes). (C) Montage images highlighting a slight increase of RhoA activity in the region of the growth cone that shows a mild collapse. **(D)** Cdc42 activity determined by ratiometric FRET live imaging. Frames were taken every second (left: generation of a ratiometric image) for 15 minutes after the stimulation (the cross indicates the position of the lipid vesicle encapsulating BSA). Insets on the right show a time serie (1 frame every 3 minutes) of the magnified region denoted by the white box in the left image. The red line shows the initial edge profile. Scale Bar: 20µm. **(E** and **F)** Montage images showing Cdc42 activation dynamics in the stimulated cell growth cones. (E) Images showing the region of interest selected for the montage (white boxes). (F) Montage images highlighting a higher activation in the growing filopodium. Intensity scales on the right in (C, F) apply to (A – F).

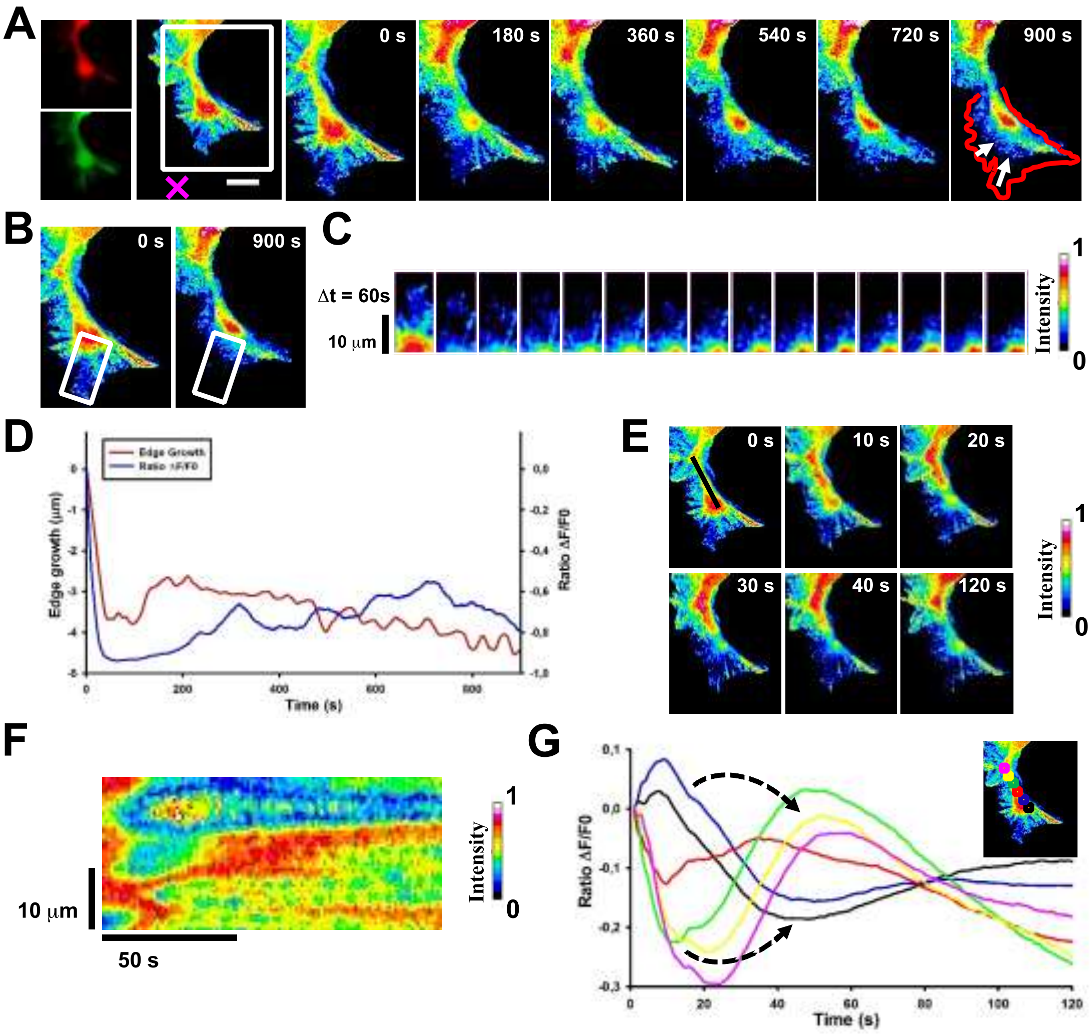

Figure S3: Cdc42 activity in a cell neurite upon stimulation with lipid vesicles encapsulating Sema3A. (A) Cdc42 activity determined by ratiometric FRET live imaging. Frames were taken every second (left: generation of a ratiometric image) for 15 minutes after the stimulation (the cross indicates the position of the lipid vesicle encapsulating Sema3A). Insets on the right show a time serie (1 frame every 3 minutes) of the magnified region denoted by the white box in the left image. Arrows indicate retraction directions. The red line shows the initial edge profile. Scale Bar: 20µm. **(B** and **C)** Montage images showing Cdc42 activity in the retracting region. (B) Images showing the region of interest selected for the montage (white boxes). (C) Montage images highlighting a decrease of Cdc42 activity in the retracting region of the cell facing the lipid vesicle filled with Sema3A. Images are taken at 60s intervals. In this case we observed a strong decrease of Cdc42 activity within 60 seconds from Sema3A delivery that proceeds simultaneously with the growth cone retraction. **(D)** Plot of average Cdc42 activity ($\Delta F/F_0$) versus edge growth from the area selected in (B). Negative values are defined as retraction. FRET ratio is represented as a blue line; edge growth is defined as a red line. **(E)** Legend as in (A), but the time span is 120 seconds. Black line indicates the area selected for kymograph analysis in **(F)** that highlights Cdc42 wave behavior. Intensity scales on the right in (C), (E), (F) apply to (A), (B), (C), (E) and (F). **(G)** Plot of average Cdc42 activity ($\Delta F/F_0$) in the regions defined by the colored squares in the inset image on the top right. The analyzed cell is the same shown in (E). Arrows indicate the temporal progression of the waves.

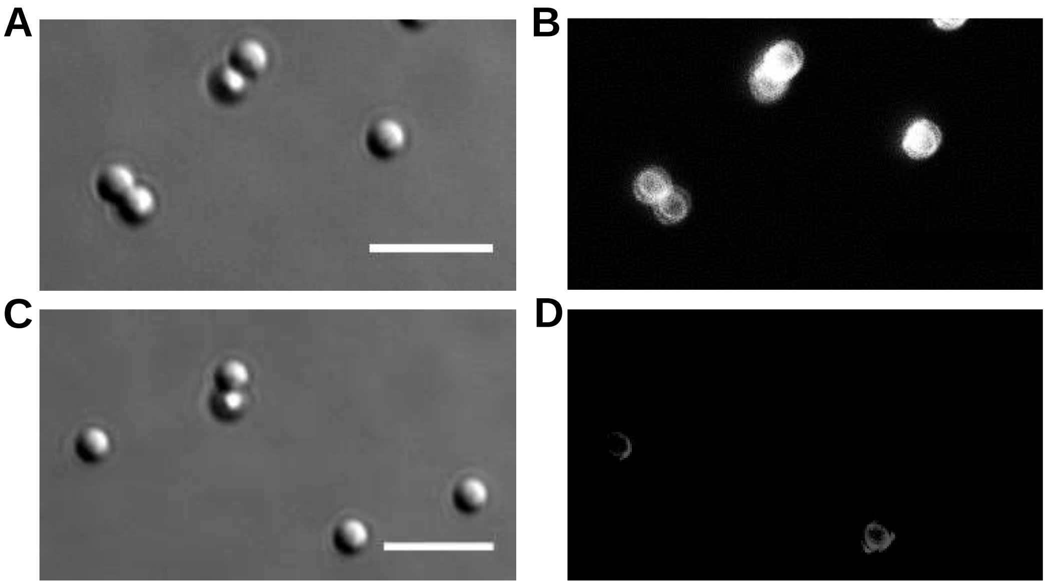

Figure S4. Sema3A-coated beads. Immunofluorescence images of Sema3A-beads (B) and of un-functionalized beads (D) incubated with the anti-Sema3A antibody following the protocol indicated in the Materials and Methods section. (A) and (C) are DIC images of the beads. Scale bar 5µm.

Supplementary Movie 1: Sema3A local stimulation leads to an activation of RhoA followed by growth cone collapse and retraction.

This movie shows the RhoA activation dynamics upon local stimulation by breaking of a lipid vescicle (magenta cross in the image) filled with 1 µM of Sema3A.
Frame interval: 4 s. Duration of original sequence: 15 min. Frames are color-coded 8 bit: warm colors represent higher activation and cold colors represent lower activation.

Supplementary Movie 2: Sema3A local stimulation induces an activation of Cdc42 in the region opposite to the stimulus

This movie shows the Cdc42 activation dynamics upon local stimulation by breaking of a lipid vescicle (magenta cross in the image) filled with 1 µM of Sema3A.
Frame interval: 4 s. Duration of original sequence: 15 min. Frames are color-coded 8 bit: warm colors represent higher activation and cold colors represent lower activation.
